# High Heat Treatment of Goat Cheese Milk. The Effect on Sensory Profile, Consumer Acceptance and Microstructure of Cheese

**DOI:** 10.3390/foods10051116

**Published:** 2021-05-18

**Authors:** Zorana Miloradovic, Nikola Tomic, Nemanja Kljajevic, Steva Levic, Vladimir Pavlovic, Marijana Blazic, Jelena Miocinovic

**Affiliations:** 1 Department of Animal Source Food Technology, Faculty of Agriculture, University of Belgrade, Nemanjina 6, 11080 Belgrade, Serbia; nemanja.kljajevic@agrif.bg.ac.rs (N.K.); jmiocin@agrif.bg.ac.rs (J.M.); 2 Department of Food Safety and Quality Management, Faculty of Agriculture, University of Belgrade, Nemanjina 6, 11080 Belgrade, Serbia; tsnikola@agrif.bg.ac.rs; 3 Department of Food Technology and Biochemistry, Faculty of Agriculture, University of Belgrade, Nemanjina 6, 11080 Belgrade, Serbia; stevalevic@gmail.com; 4 Department for Mathematics and Physics, Faculty of Agriculture, University of Belgrade, Nemanjina 6, 11080 Belgrade, Serbia; vlaver@agrif.bg.ac.rs; 5 Institute of Technical Sciences of Serbian Academy of Sciences and Arts, Knez Mihailova 35/IV, 11000 Belgrade, Serbia; 6 Department of Food Technology, Karlovac University of Applied Sciences, Trg J.J. Strossmayera 9, 47000 Karlovac, Croatia; marijana.blazic@vuka.hr; 7 University College Aspira, Mike Tripala 6, 21000 Split, Croatia

**Keywords:** brined cheese, product development, preference mapping, mean drop analysis, scanning electron microscopy

## Abstract

Goat cheeses from high heat treated milk (HHTM: 80 °C/5 min (E1) and 90 °C/5 min (E2)), could be regarded as new products, compared to their analogues made from commonly pasteurized milk (65 °C/30 min (C)). Descriptive analysis and consumer tests with a hedonic scale and JAR scale were part of the product development process. The use of scanning electron microscopy enabled deeper insight into the flavor and texture of the cheeses. In all cheese variants, goaty flavor was mildly pronounced. Young HHTM cheeses also had a pronounced whey and cooked/milky flavor. Consumers found such flavor ‘too intensive’. Unlike the control variant, HHTM cheeses were not described as ‘too hard’. Such improvement in texture was found to be a result of fine, highly branched microstructure, sustained over the course of ripening time and highly incorporated milk fat globules inside the cheese mass. Cluster analysis showed that the largest group of consumers (47.5%) preferred E2 cheese. Although consumers found that most of the cheeses were ‘too salty’, this excess did not decrease their overall acceptance. Neither microstructure analysis nor descriptive sensory analysis of goat white brined cheeses produced from high heat treated milk has been done before.

## 1. Introduction

The popularity of goat cheeses is continuously increasing, mostly for reasons of health, nutritional value, the associated images of “craft“ and “natural“, but also because of their special flavor [1,2,3].

High heat treatment of milk (HHTM) consists of heating regimes with temperatures higher than common pasteurization (65 °C/30 min or 72 °C/15 s). During HHTM, modification of the structural organization of milk proteins occur, salt balance change, enzymes are being inactivated and proteins denatured [4]. All mentioned changes cause numerous effects on the final dairy products. Some of them are beneficial, such as the extended shelf-life of dairy beverages [5], the improved physical properties of fermented dairy products [4] and the enhanced cheese yield [6,7]. Aside of beneficial effects, HHTM causes a negative effects on rennet-coagulation properties of cow milk: an increase of gelation time, reduction of curd firmness and prolongation of set-to-cut time [8]. However, in the case of goat milk, due to the specific mineral composition and casein micelle structure, rennet-coagulation properties are much less impaired. Hence, high heating regimes have been successfully applied for goat rennet cheese yield enhancement [9]. Such a simple method leading to higher profitability is particularly significant since almost all goat cheese production is done at small scale [10].

The quality of cheeses produced from HHTM differs significantly from the analogue cheeses made from commonly pasteurized milk [9]. Therefore, they could be regarded as new products. In the development of any new product, sensory evaluation is considered to be the most useful tool for achieving, primarily, quality improvement and for ensuring market success [11].

The sensory quality of HHTM cheeses (80 °C/5 min and 90 °C/5 min) in the early (3 days), mid (10 days) and late (40 days) ripening stages has been evaluated by trained panelists [9]. All the cheeses had very-good to excellent quality. Determination of sensory quality focuses mainly on defect identification, and compares samples to an ideal prototype. However, consumer evaluation of a product often differs from what experts judge to be good or bad quality. In consequence, it was suggested decades ago that harmonization should exist between consumer opinion and expert judgement [12].

Cheese acceptability and its quality depend greatly on its flavor. Goat milk cheeses are characterized by a unique flavor, often robust and very complex, that makes them different from cow milk cheeses [13]. Such a unique flavor attracts highly demanding specialty cheese consumers [14] and satisfying their tastes is a great challenge for producers. In order to help producers, and to close the gap that exists in scientific research in goat cheese sensory analysis in general [13], this present study conducted a descriptive sensory analysis to determine the flavor profile of goat cheeses produced from HHTM and from commonly pasteurized milk, in different ripening stages. Descriptive sensory analysis is a costly and time consuming method, and has rarely been done for goat cheese [1,15]. Another aim of this study was to test how consumers perceive the said cheeses. Consumer tests, along with the mean drop analysis could well determine the direction for future product optimization [16]. In order to gain a deeper understanding of what causes the differences in flavor and in texture of the cheeses analyzed in the present study, scanning electron microscopy was conducted. Neither the microstructure nor the descriptive sensory analysis of goat white brined cheeses produced from high heat treated milk has been done before. We assume that the findings of the present study would be useful for new cheese variety development, and for all the future studies concerning cheeses of different kinds, produced from high heat treated goat milk.

## 2. Material and Methods

### 2.1. Cheese Manufacture

Cheese making trials were carried out on three occasions at the Pilot Plant of the Faculty of Agriculture, University of Belgrade, Serbia. For each trial, 60 L of milk (dry matter 10.30 ± 0.04; total proteins 2.57 ± 0.10; total milk fat 2.75 ± 0.15; pH 6.63 ± 0.04) was divided into three 20 L lots. The lots were subjected to different heat treatments: 65 °C/30 min (control cheese C), 80 °C/5 min (E1) and 90 °C/5 min (E2), and three cheese variants were made according to the identical procedure. Starter inoculation was carried out at 31 °C with 0.005% (*w*/*v*) of mesophilic starter culture e Lyofast MWO 030 (ClericiSacco Group, Cadorago, Italy). The milk was allowed to ripen until the pH value reached 6.5. Then, at 31 °C, 0.02% (*w*/*v*) CaCl_2_ and calf rennet Caglificio Clerici (Clerici-Sacco Group, Cadorago, Italy) consisting of 94% chymosin and 6% pepsin were added. The amount of added rennet was 0.2 g per 10 L of milk. After 45 min of rennet coagulation, the curd was cut into cubes (5 cm sides), left to rest for 15 min, and carefully transferred to rectangular molds (30 cm × 40 cm) with cotton cloth, and allowed to drain for 10 min. The curd was pressed for 1 h with 2 kg of weight per kg of curd, and then, for another hour, with 4 kg kg ^−1^ of curd, at room temperature. After pressing (2 h in total), curd was cut into blocks (15 cm × 7 cm × 2 cm). The curd blocks were dry salted with 3% (by the weight of curd blocks) fine grain iodized NaCl (So Produkt, Stara Pazova, Serbia), placed into individual plastic containers, and stored at 23–25 °C for 24 h. After 24 h the plastic containers were filled with 6% (*w*/*w*) NaCl brine, previously adjusted to pH 5.0 with lactic acid and pasteurized at 80 °C/10 min and then cooled to room temperature. The ratio of brine volume to cheese weight was 1:2. The containers were sealed and the cheeses were ripened at 13–15 °C for 40 days.

Cheese samples were taken in the early (3 days), mid (10 days) and late (40 days) ripening stages for sensory and microstructure analysis. In total, there were nine cheese samples. Their labels, and basic characteristics were given in Table 1.

### 2.2. Sensory Analysis

The descriptive sensory analysis and the consumer acceptance testing were performed in the laboratory for sensory testing at the University of Belgrade—Faculty of Agriculture. The cheese samples were labeled with three random digit codes. Prior to testing, cheese blocks were removed from brine and tempered at 20 °C until their surfaces dried. During evaluation, the assessors used water and unsalted crackers against the aftertaste effect.

#### 2.2.1. Descriptive Sensory Analysis

For the purpose of descriptive analysis, all nine cheese samples (Table 1) were served at the same time, and the evaluation was performed in two trials. The sensory panel consisted of 8 members (4 man and 4 women), aged between 23 and 45 years, all staff members and master students of the University. Nine 2-h training sessions were performed over a five week period, focused on identifying and analyzing the cheese flavor components of interest. The training included: generating sensory attributes; fixing the minimum and maximum for each attribute; practicing the usage of intensity scales for evaluation of sensory attributes of different types of goat cheeses from the market, including white brined cheeses.

The panelists determined the list of flavor attributes using a slightly modified lexicon for goat cheese, adapted from Carunchia Whetstine, et al. [17]. The defined attributes together with definitions and reference standards are presented in Table 2. Flavor intensity was evaluated using a 15 cm line scale.

#### 2.2.2. Consumer Testing

The consumer testing was performed by 50 voluntary participants, consisting of students and university staff, friends and relatives (18–70 years old). They were selected randomly and were checked to ensure they did not have an aversion to goat cheese.

The cheese samples were evaluated using the nine-point hedonic scale, in terms of liking ‘the product as a whole’, ‘the odor’, ‘the flavor’ and ‘the texture’. ‘Extreme dislike’ was scored with 1 and ‘extreme like’ with 9, and neutral attitude was 5.

Along with the hedonic scale, the nine-point JAR scale (just-about-right) was used for the intensity of sour taste (not sour enough/JAR/too sour), for the salty taste (not salty enough/JAR/too salty), the overall intensity of flavor (not intensive enough/JAR/too intensive), the hardness (to soft/JAR/too hard) and the greasiness (to dry/JAR/too greasy).

### 2.3. Scanning Electronic Microscopy (SEM)

The samples for SEM analysis (approximately5 mm × 5 mm × 15 mm) were cut from the interior of cheese blocks of C, E1 and E2 cheese variants after 3, 10 and 40 days of ripening. Samples were defatted and fixed as in the study of Miloradovic et al. [10] critical point dried with liquid carbon dioxide, using a K850 Critical Point Drier (Quorum Technologies, Laughton, UK). Dried fractured rennet gel samples were attached to stubs and sputter coated with gold (50 nm) for 100 s at 30 mA (Sputter Coater BAL-TEC SCD 005, Scotia, New York, NY, USA). Samples were examined with a JEOL JSM 6390LV scanning electronic microscope (JEOL, Tokyo, Japan). The images were recorded at both 3500× and 5000×, but only 3500× images were included in the present paper.

### 2.4. Statistical Analysis

#### 2.4.1. Descriptive Data and PREFMAP

Individual descriptive data were standardized prior to multivariate and univariate analysis of variance (MANOVA and ANOVA, respectively). One way MANOVA, with ‘cheese sample’ as a fixed factor, was conducted to test the significance of the multivariate effect. The standardized descriptive data were then subjected to three-way ANOVA, with ‘cheese sample’ as a fixed factor (main effect), and ‘panelists’ and ‘replications’ as random factors.

In order to perform generalized Procrustes analysis (GPA), raw descriptive data were divided into 16 personal construct grids (8 assessors × 2 replications). The consensus data matrix obtained by GPA (9 rows/cheese samples and 9 columns/sensory attributes) was further analyzed by principal component analysis (PCA).

#### 2.4.2. Consumer Data

Following the PCA, the technique called external preference mapping (PREFMAP) was applied as described by Tomic, et al. [18]. The raw hedonic data were regressed against the principal components extracted by the PCA performed on descriptive data. The consumers (*n* = 50) were divided into three clusters based on their similar opinions about cheese acceptance. Mean values for regression coefficients for identified clusters were included in the PCA graphs.

The hedonic data were analyzed by three-way ANOVA, with three fixed factors: ‘clusters’, ‘heat treatment’ and ‘ripening time’. For the purpose of mean comparisons, the LSD test was performed at the level of significance α = 0.05.

The raw JAR-data were classified into three groups: below JAR (scores 1,2,3), JAR (scores 4,5,6) and above JAR (scores 7,8,9). In order to determine the extent to which the deviation from just-about-right affected the overall acceptability of cheeses, mean drop analysis was performed as described by Schraidt [19]. Raw JAR scores were grouped into three categories as follows: 1, 2 and 3 ¼ ‘below JAR’; 4, 5 and 6 ¼ ‘at JAR’; 7, 8 and 9 ¼ ‘above JAR’. Then the mean overall hedonic rating was calculated for each category. Mean drops were calculated by subtracting the mean liking of each non-JAR category from the mean of the JAR category. The JAR categories overall hedonic means were compared by an ANOVA and Tukey’s HSD test. Minimum percentage skew for ‘Not Just Right’ (the cutoff) was set at 20% of the total consumer panel.

#### 2.4.3. Software

Idiogrid version 2.4 was used for GPA and PCA, and also for data standardization, while SPSS 17.0 was used for the rest of the calculations. Statistical significance level was set at 0.05.

## 3. Results and Discussion

### 3.1. Descriptive Sensory Analysis and PREFMAP

The raw data from the nine sensory attributes discriminated among cheese variants were subjected to GPA. The results showed a consensus proportion of 0.74 (74%; statistically significant at α = 0.05) indicating a relatively strong agreement among accessors, including replicates. Consensus data were further subjected to PCA. The first three principal components were retained for being in agreement with the Kaiser criterion. The three PCs explained 89.25% of the variance for the raw variables (sensory attributes): PC 1 = 38.88%; PC2 = 29.60% and PC3 = 20.78%.

**Figure 1:**
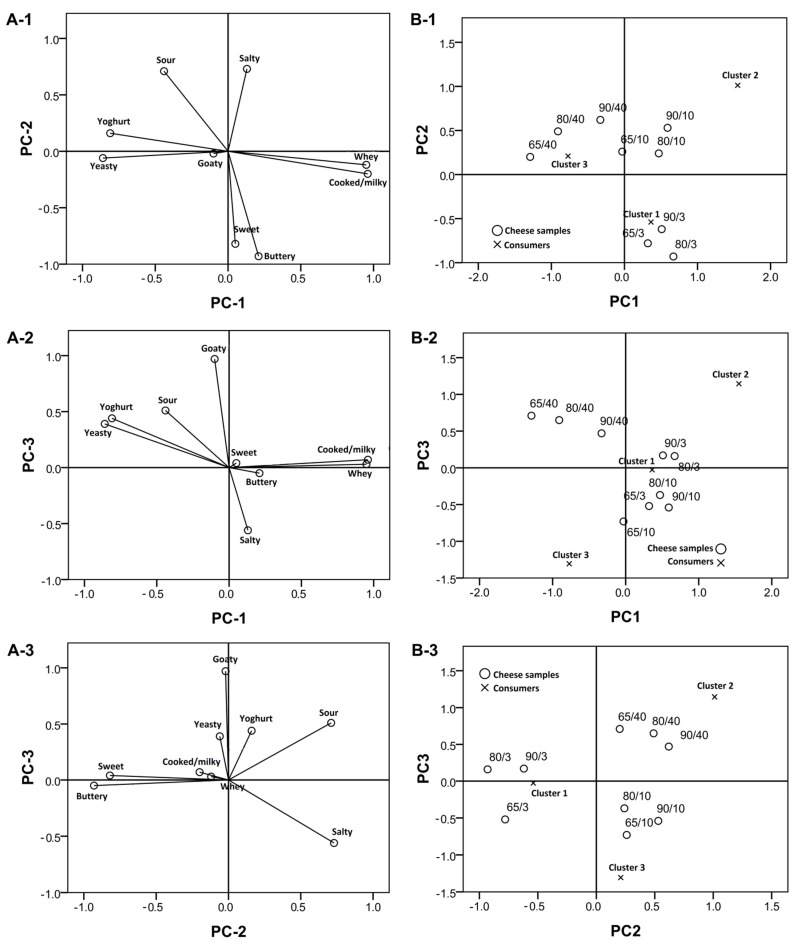
Attribute loadings (**A-1**–**A-3**) and cheese scores/clusters (**B-1**–**B-3**). The first three principal components obtained by the generalized Procrustes analysis on descriptive data (8 assessors/2 replications) of goat cheeses produced from milk heated to 65 °C/30 min at the early (3 days), mid (10 days) and late (40 days) stage of ripening (65/3, 65/10 and 65/40, respectively), milk heated to 80 °C/5 min at the early, mid and late stage of ripening (80/3, 80/10 and 80/40, respectively) and milk heated to 90 °C/5 min, at the early, mid and late stage of ripening (90/3, 90/10 and 90/40, respectively).

**Figure 2:**
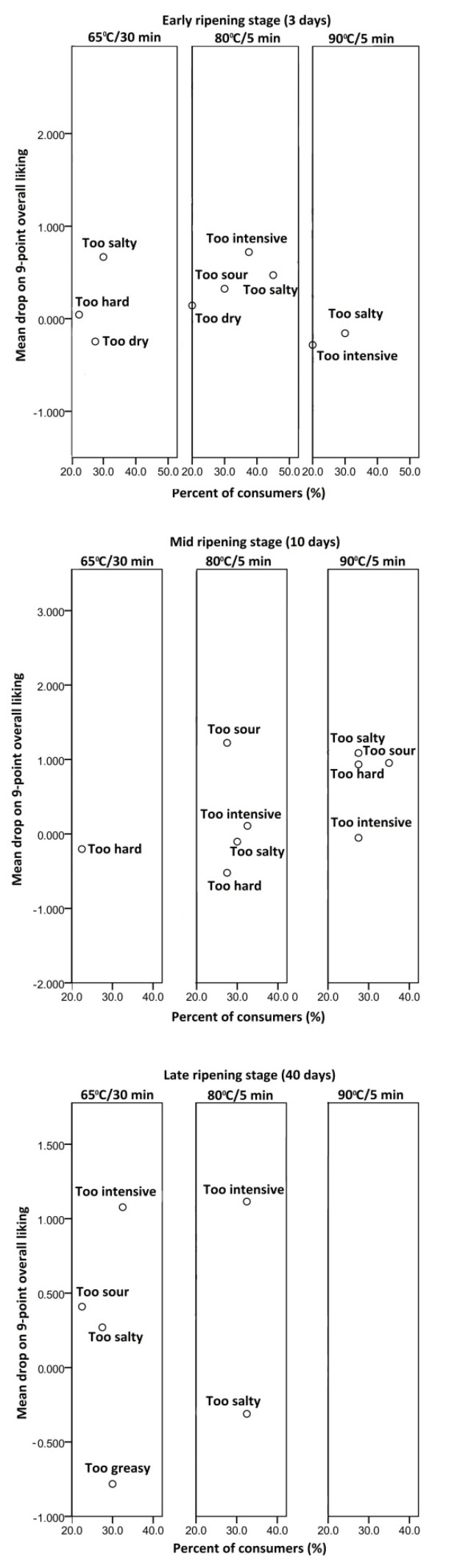
Mean drop analysis of goat cheeses produced from milk heated to 65 °C/30 min, 80 °C/5 min and 90 °C/5 min, in the early (3 days), mid (10 days) and late (40 days) stage of ripening.

**Figure 3:**
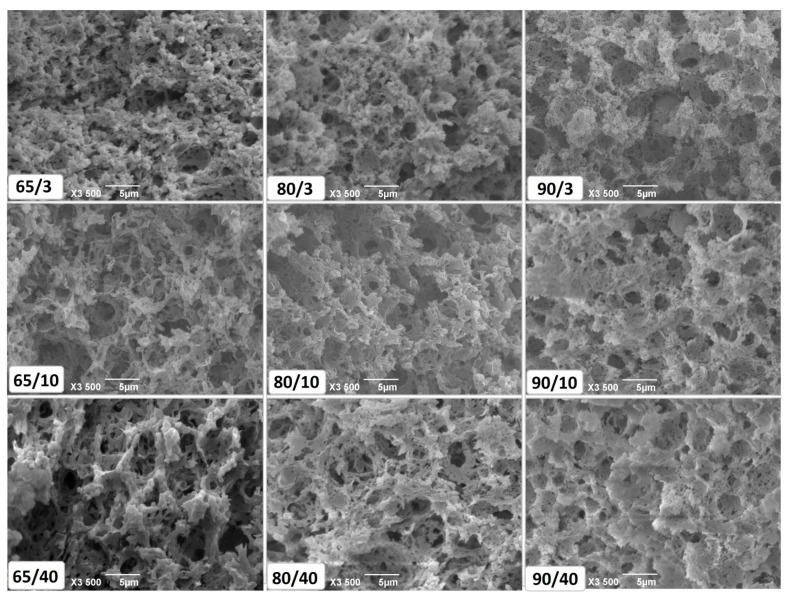
Microstructure of goat cheeses produced from milk heated to 65 °C/30 min, 80 °C/5 min and 90 °C/5 min, in the early (3 days) ripening stage (65/3, 80/3 and 90/3), mid (10 days) ripening stage (65/10, 80/10 and 90/10) and late (40 days) ripening stage (65/40, 80/40 and 90/40).

Figure 1(A-1–A-3) represents sensory attributes correlated to principal components (the loadings plots), while the charts B-1–B-3 on the same figure show cheese samples and consumer-clusters scores of the three extracted PCs (the scores plots).

The results show that the goaty flavor was only mildly pronounced in all the samples analyzed, presumably as a result of good hygiene and careful handling of raw milk. Such a practice limits lipolysis and, consequently, the goaty taste, as well [13,20].

Heat treatment of milk destroys the native goat milk lipase [13] and could explain why the intensity of goaty flavor did not increase at the late stage of ripening. It should also be pointed out that HHT of milk did not cause an increase of goaty flavor intensity, which could be seen as an advantage since some consumers find it undesirable [13].

It was expected that HHTM cheeses would have pronounced whey and cooked flavors, and this presumption was correct in relation to the early and mid-ripening stages. In the late ripening stage, as often occurs [13], these flavor attributes were clouded by the creation of more pronounced flavors. In the mid ripening stage, cheeses were characterized by a pronounced saltiness that dominated over all other flavor attributes. After 40 days of ripening, all cheese variants, and especially 65/40, had a sour, yoghurt and yeasty flavor.

Consumers’ hedonic scores were regressed against the three PCs. Three clusters of consumers were identified by K-means clustering, each one with at least 20% of respondents: Cluster 1 (47.5%), Cluster 2 (32.5%) and Cluster 3 (20%).

The flavor of goat cheese has always been debatable. Some consumers prefer neutral varieties that resemble cow cheeses, while others particularly appreciate typical goaty flavor [1]. This study showed that the largest group of consumers within Cluster 1 (47.5%) was more attracted to mild-flavored young cheeses (Table 3), characterized by such attributes as sweet and buttery, cooked/milky and whey flavors. This group also favored the cheese from milk heated at 90 °C/5 min in all three ripening stages. Since that was an experimental cheese variant, it was surprising that the largest group was accepting a flavor they had never tasted before. According to that, they could be labeled as innovative consumers [14]. On the other hand, Cluster 3 (20%) preferred cheese from commonly pasteurized milk (65 °C/30 min). In terms of ripening stage, they favored 10 days ripened cheeses, which were plotted in the range of the least intensive flavors and a dominating salty taste (Figure 1). Such cheeses are exactly the types that could be found at local markets, so this group could be labeled as neophobic consumers—the ones who prefer the common flavors and are not willing to try new ones [21]. Cluster 2 (32.5%) was in between, with high scores given to both control cheese and to experimental ones, hence it could be assumed that this group comprised agreeable consumers [22] or simply goat cheese appreciators.

The lack of superscript or the similar superscript indicate that values within the row did not differ significantly (α > 0.05).

### 3.2. Mean Drop Analysis

The analysis revealed that there was not a single large group (accounting for at least 20% of consumers) with a significant mean drop of overall acceptability, considering all cheese samples (Figure 2). This indicated that although the groups of consumers had certain objections to particular sensory characteristics, this did not diminish overall acceptability of the cheese.

For example, the ‘too salty’ evaluation was given to almost all cheese variants in all ripening stages. However, white brined cheese is traditionally consumed in Serbia with salads (Srpska, Šopska) or in bakery products (Burek, Cheese pie) [23]. Accordingly, it could be assumed that the excessive saltiness of cheese would be well tolerated by consumers when used as an ingredient. However, an earlier attempt to reduce the salt content in HHTM goat white brined cheese resulted in decreased consumer acceptability [24]. A possible explanation could be that Serbian consumers are not aware of the risks associated with excessive salt intake.

The cheese variant 90/40 was rated ‘just about right’ for saltiness and for all other proposed characteristics, suggesting that for this heat treatment of milk, 40 days of ripening had a good effect on the flavor balance. Excepting the 90/40, consumers perceived the flavor of HHTM cheeses as ‘too intensive’. This indicates that consumers did notice the different flavor components that appeared in cheeses as a result of high heat treatment of milk. However, as was the case with the earlier example of excessive saltiness, ‘too intensive’ flavor was also well tolerated by consumers. This positive approach to new flavor experiences was typical of the innovative consumer type, which makes up almost a half of the sample (47.5%), determined by cluster analysis and already discussed in Section 3.1.

All the cheeses after 10 days of ripening were characterized by consumers as being ‘too hard’. In the early stage of ripening, 65/3 was also described as ‘too hard’, but no such remark was given for 80/3 and 90/3. After being matured for 40 days, neither cheese received such an evaluation. Rubbery texture has already been reported for the young C variant, while the young HHTM cheese resembled mature cheese in terms of texture [9]. All this might suggest that while cheese C needs ripening time for development of an acceptable texture, HHTM cheeses could be consumed both when young and when in late ripening stages.

### 3.3. Microstructure

It has already been reported that a coarse microstructure occurs when acid-coagulated cheeses are produced from unheated milk. On the other hand, the microstructure of the analogue cheeses made from high heat treated milk is “fine“, highly branched [25]. The same pattern has been observed for cheeses in this study (Figure 3). Although coagulated by rennet, the presence of incorporated whey proteins and the low pH values of E1 and E2 cheeses [9] provide the elements for an acid coagulated cheese microstructure. A fine, highly branched microstructure made them softer than the control variant at the early ripening stage—not ‘too hard’, as C was characterized by consumers.

The microstructure of rennet gel (45 min after the addition of rennet) from milk heated to 80 °C/5 min showed higher level of similarity to gel from commonly pasteurized milk (65 °C/30 min) than to gel from milk heated to 90 °C/5 min [10]. After cheese production, a number of processes occur simultaneously, caused by fermentation/acidification. Due to a pH decrease below 5.00, and with the presence of whey proteins [9], the E1 variant developed a fine branched protein structure, more similar to E2 then to C.

The state in which milk fat is incorporated, plays an important role in the sensory properties of cheese [26]. Cheeses can contain small fat globules (2 µm in diameter), aggregates or large fat areas—pools of fat enveloped in the protein matrix [27]. Following high heat treatment, milk fat globule membrane (MFGM) components interact with milk proteins [26]. Spherical void spaces, found in the HHTM cheese microstructure (especially E2), indicate that fat globules are an integral part of the protein matrix. In the control cheese variant, milk fat appeared to be, predominantly, just enveloped in the protein matrix together with serum. Only a few spherical void spaces were notable in the micrograph. When small fat globules are incorporated in cheese, its structure is not easily deformed or ruptured [27]. This could explain the stable texture (fracture hardness) present in HHTM cheeses, as reported earlier [9]. 

During the ripening of semihard cheeses, the casein matrix forms the structure that is more homogenous, with smaller void spaces [27]. However, the present study showed that the opposite is the case for white brined cheeses. During the ripening, the surrounding brine protrudes into serum pockets and breaks protein chains already weakened by proteolysis. It could be seen that, with a higher level of proteolysis in the control cheese [9], larger void spaces were occurring. In consequence, in the later stages of ripening, fat could be lost in surrounding brine. Therefore, high heat treatment of milk could be used as a method for keeping the fat highly integrated into the cheese matrix during its ripening in brine.

## 4. Conclusions

High heat treatment of milk, and especially 90 °C/5 min, could be considered an improvement in the making process of goat white brined cheeses. On top of the yield enhancement that was reported earlier, it provided a more stable microstructure that keeps milk fat integrated into the cheese body during ripening in brine. Furthermore, it produced a fine, highly branched microstructure that eliminated a ‘too hard’ evaluation by consumers of the control cheese in its early ripening stages.

The resulting cheese was recognized as a product with high potential for further development, likely to be well accepted at the market, since the largest subgroup of consumers (47.5%) preferred the E2 cheese to the other two varieties. Flavor profiles of cheese varieties, determined in the present study, could serve as the basis for optimization of cheese quality in future product development.

It is also important to point out that, although consumers were well aware that most of the cheeses were ‘too salty’, the drop in acceptance was not significant. The reason could be the Serbian tradition of consuming white brined cheeses mostly as an ingredient. However, it could also mean that they are just not aware of the risks associated with excessive salt intake. A survey study should be performed in order to clear up that issue. Additionally, consumer awareness of the risks associated with excessive salt intake should be raised.

It could be concluded that white brined cheeses produced from high heat treated goat milk are not only more profitable to the producer but have a higher sensory acceptability among consumers.

## Figures and Tables

**Table 1:** Cheeses produced with milk heated to 65 °C/30 min (C), 80 °C/5 min (E1) and 90 °C/5 min (E2), at the early (3 days), mid (10 days) and late (40 days) stage of ripening; Labeling and basic characteristics.

Heat Treatment	Ripening Stage (Days)	Label	DM ^1^ (%)	TP ^1^(%)	MF ^1^(%)	pH	NaCl (%)
65 °C/30 min	3	65/3	42.38 ± 1.22	17.35 ± 0.44	18.41 ± 0.91	5.02 ± 0.11	1.96 ± 0.25
	10	65/10	40.34 ± 1.31	16.35 ± 0.87	17.67 ± 1.53	4.94 ± 0.11	2.71 ± 0.37
	40	65/40	38.16 ± 3.82	16.03 ± 1.32	17.50 ± 2.64	5.00 ± 0.07	3.29 ± 0.53
80 °C/5 min	3	80/3	38.95 ± 1.12	15.25 ± 0.32	17.98 ± 0.86	4.71 ± 0.16	1.72 ± 0.32
	10	80/10	39.93 ± 1.52	16.17 ± 0.96	18.00 ± 0.87	4.59 ± 0.04	2.58 ± 0.26
	40	80/40	39.84 ± 0.99	15.63 ± 0.90	18.58 ± 0.52	4.50 ± 0.08	3.09 ± 0.46
90 °C/5 min	3	90/3	38.05 ± 0.66	14.78 ± 1.36	18.20 ± 0.63	4.76 ± 0.06	1.75 ± 0.51
	10	90/10	38.76 ± 0.43	14.94 ± 0.33	18.17 ± 0.28	4.47 ± 0.10	2.67 ± 0.45
	40	90/40	39.49 ± 1.38	15.86 ± 0.90	19.00 ± 1.73	4.49 ± 0.12	3.39 ± 0.92

Adapted from Miloradovic et al. [9]. **^1^** DM—dry matter; TP—total protein; MF—milk fat.

**Table 2:** Goat cheese taste attributes and reference standards.

Term	Definition	Reference Standards
Buttery	Aromatics associated with butter	Butter “Meggle”, Kragujevac
Cooked/milky	Aromatics associated with cooked milk (mix of sweet and caramelized)	Pasteurized milk “Imlek”, Padinska Skela, 2.8% milk fat, cooked at 85 °C/10 min
Goaty	Aromatics associated with wet animal hair	Raw goat milk
Whey	Aromatics associated with cow’s whey	Fresh cow whey
Yeasty	Aromatics associated with baker’s yeast	Fresh yeast, “Vrenje”, Belgrade, dissolved in water (3%)
Yoghurt	Aromatics associated with yoghurt	Yoghurt, “Imlek”, Padinska Skela, 2.8% milk fat
Salty	Fundamental taste sensation elicited by salts	NaCl water solution (1%)
Sour	Fundamental taste sensation elicited by citric, acetic, apple acids	Citric acid, water solution(0,08%)
Sweet	Fundamental taste sensation elicited by saccharose	Saccharose, water solution (1%)

Adapted from Carunchia Whetstine et al. [17].

**Table 3:** Consumer hedonic test scores for goat cheeses produced from milk heated by three different treatments, in the early, mid and late ripening stage.

Ripening Stage	Heat Treatment	Hedonic Score	Consumers (*n* = 80)		
			Cluster 1 (47.5%)	Cluster 2 (32.5%)	Cluster 3 (20%)
3 days	65 °C/30 min	ODOUR	7.25 ± 1.77	7.84 ± 1.41	7.28 ± 2.43
(early)		TASTE	6.95 ± 1.73	7.84 ± 1.14	7.43 ± 2.23
		TEXTURE	6.75 ^a^ ± 1.74	8.23 ^b^ ± 1.01	7.14 ^ab^ ± 1.86
		OVERALL	6.85 ^a^ ± 1.35	8.38 ^b^ ± 0.87	8.00 ^ab^ ± 1.29
	80 °C/5 min	ODOUR	6.85 ± 2.23	7.30 ± 1.80	5.71 ± 2.81
		TASTE	7.25 ± 1.62	7.77 ± 1.36	6.43 ± 2.64
		TEXTURE	6.60 ± 1.88	7.31 ± 1.80	6.28 ± 2.69
		OVERALL	7.20 ± 1.58	7.61 ± 1.85	6.71 ± 1.25
	90 °C/5 min	ODOUR	7.85 ^b^ ± 2.13	7.92 ^b^ ± 1.12	5.57 ^a^ ± 1.51
		TASTE	8.00 ^b^ ± 1.52	8.46 ^b^ ± 0.97	5.86 ^a^ ± 1.86
		TEXTURE	7.55 ^b^ ± 1.82	8.00 ^b^ ± 1.00	5.00 ^a^ ± 2.00
		OVERALL	8.35 ^b^ ± 0.93	8.23 ^b^ ± 0.83	6.43 ^a^ ± 1.81
10 days	65 °C/30 min	ODOUR	7.45 ± 1.90	6.69 ± 2.18	7.28 ± 1.70
(mid)		TASTE	6.85 ^a^ ± 1.79	8.08 ^b^ ± 0.76	7.57 ^ab^ ± 1.81
		TEXTURE	6.90 ± 1.80	7.54 ± 1.51	7.57 ± 2.57
		OVERALL	6.75 ^a^ ± 1.29	7.54 ^ab^ ± 1.33	8.57 ^b^ ± 0.79
	80 °C/5 min	ODOUR	7.35 ± 1.46	7.31 ± 1.55	8.00 ± 1.15
		TASTE	7.45 ± 1.54	7.61 ± 1.61	7.28 ± 1.50
		TEXTURE	7.00 ^ab^ ± 1.81	8.15 ^a^ ± 0.90	6.14 ^b^ ± 3.08
		OVERALL	7.25 ± 1.45	7.23 ± 1.69	7.71 ± 1.11
	90 °C/5 min	ODOUR	7.90 ^a^ ± 1.29	6.23 ^b^ ± 2.35	7.28 ^ab^ ± 1.25
		TASTE	8.35 ^a^ ± 0.99	7.23 ^b^ ± 2.01	7.71 ^ab^ ± 1.11
		TEXTURE	7.95 ± 0.89	7.31 ± 1.60	6.86 ± 2.41
		OVERALL	8.30 ^a^ ± 0.80	7.08 ^b^ ± 2.56	8.28 ^ab^ ± 0.95
40 days	65 °C/30 min	ODOUR	5.15 ± 2.78	4.92 ± 2.47	5.00 ± 2.16
(late)		TASTE	6.20 ± 2.04	6.61 ± 1.89	7.28 ± 0.95
		TEXTURE	6.00 ± 2.29	6.38 ± 1.71	7.28 ± 1.60
		OVERALL	6.15 ^a^ ± 1.95	6.85 ^ab^ ± 1.21	7.71 ^b^ ± 1.11
	80 °C/5 min	ODOUR	6.05 ± 2.44	6.77 ± 1.54	5.71 ± 2.36
		TASTE	6.55 ± 2.04	7.23 ± 1.42	6.57 ± 2.23
		TEXTURE	6.25 ± 2.20	6.69 ± 1.44	5.86 ± 2.61
		OVERALL	6.80 ± 1.74	7.15 ± 1.57	7.28 ± 1.25
	90 °C/5 min	ODOUR	7.55 ^a^ ± 1.70	7.31 ^ab^ ± 1.75	5.57 ^b^ ± 2.44
		TASTE	8.40 ± 0.94	7.85 ± 1.28	7.86 ± 0.90
		TEXTURE	7.85 ± 1.63	7.00 ± 1.68	7.28 ± 2.14
		OVERALL	8.40 ± 0.82	7.69 ± 1.65	7.57 ± 0.98

Values marked with the different superscript within row are significantly different (α < 0.05).

## Data Availability

The data presented in this study are available on request from the corresponding author.
